# Baths and Bathing

**Published:** 1877-05

**Authors:** 


### BATHS AND BATHING.

A cold bath is in water under seventy degrees Fahrenheit, a warm one, is over ninety. A warm bath in pure water de bilitates, in salt water, strengthens.

